# ﻿Three new species of *Candolleomyces* (Agaricomycetes, Agaricales, Psathyrellaceae) from the Yanshan Mountains in China

**DOI:** 10.3897/mycokeys.88.81437

**Published:** 2022-04-13

**Authors:** Hao Zhou, GuiQiang Cheng, XiMei Sun, RuiYi Cheng, HongLiang Zhang, YanMin Dong, ChengLin Hou

**Affiliations:** 1 College of Life Science, Capital Normal University, Beijing, 100048, China Capital Normal University Beijing China; 2 Beijing Songshan National Nature Reserve Management Office, Beijing 102115, China Beijing Songshan National Nature Reserve Management Office Beijing China

**Keywords:** molecular systematics, new taxon, *Psathyrellaceae*, taxonomy

## Abstract

Three new species, *Candolleomycesincanus*, *C.subcandolleanus* and *C.yanshanensis*, were found and described from Yanshan Mountains in China. The identification is based on morphological observation combined with phylogenetic analysis of ITS-LSU-*Tef1α*-*TUB2*. This study enriched the species diversity of *Candolleomyces* in Yanshan Mountains and provided important data support for the systematic study of *Candolleomyces* in the future.

## ﻿Introduction

*Candolleomyces* Wächter & A. Melzer was established in 2020, belonging to Basidiomycota, Agaricomycetes, Agaricales, Psathyrellaceae (Wächter and Melzer 2020). In a previous study, this genus was subordinate to *Psathyrella* (Fr.) Quél. (1872) and molecular sequence data have improved understanding of relationships of *Psathyrella* species (Hopple and Vilgalys 1999; Moncalvo et al. 2002; Matheny et al. 2006). However, the combination analysis of the ITS and LSU regions showed that the delimitation of some species within *Psathyrella* are still unclear (Larsson and Örstadius 2008). In more recent studies, multi-gene loci (for example, ITS, LSU, *Tef1α* and *TUB2*) became the main methods for identification of *Psathyrella* (Wang and Bau 2014; Yan and Bau 2017, 2018a, 2018b, 2021; Yan 2018; Yan et al. 2019).

In previous studies of *Psathyrella*, there are approximately 100 taxa lacking pleurocystidia, but this feature has not been used as a key distinguishing feature (Fries 1838; Smith 1972; Kits van Waveren 1985; Örstadius and Kundsen 2012; Battistin et al. 2014). Based on extensive specimen collection, morphological studies and phylogenetic analyses, *Candolleomyces* has been separated from *Psathyrella* as a new genus and it differs from *Psathyrella* s.s. in lacking pleurocystidia. (Wächter and Melzer 2020).

Currently, there are 25 recognised species in *Candolleomyces* in the Index Fungorum website (http://www.indexfungorum.org, until Jan. 2022) and 10 species were reported in China (Yan 2018; Bau and Yan 2021).

Yanshan Mountains are located in North China and have a warm temperate continental monsoon climate, with higher plant diversity. The dominant plants include *Quercus* spp., *Betula* spp., *Abies* spp. and *Pinustabuliformis* Carr. et al. (Wang et al. 2021). There is no information about *Candolleomyces* as yet. In this study, based on morphological characters and the phylogenetic analyses, three new species of *Candolleomyces* from Yanshan Mountains in China are described.

## ﻿Materials and methods

### ﻿Morphological studies

Collections were obtained and photographed in the field from Yanshan Mountains in China from 2017 to 2020. The collected specimens were dehydrated with a dryer (Dorrex) at 50 °C and the specimens were deposited in the Herbarium of the College of Life Science, Capital Normal University, Beijing, China (**BJTC**). Macroscopic characters were recorded from specimens. Microscopic characters were observed in thin sections of specimens mounted in 3% potassium hydroxide (KOH) or sterilised water. The shape and size of microscopic structures were observed and noted using a light microscope [Olympus DP71, Tokyo, Japan]. The measurements and Q values are given as (a)b–c(d), in which “a” is the lowest value, “b–c” covers a minimum of 90% of the values and “d” is the highest value. Q stands for the ratio of length and width of a spore (Bau and Yan 2021). Nomenclatural details were submitted to the MycoBank. In this study, the morphological colour comparison was compared to the reference website colorhexa (https://www.colorhexa.com).

### ﻿DNA extraction PCR amplification and sequencing

DNA extraction was achieved by the M5 Plant Genomic DNA Kit [Mei5 Biotechnology, Co., Ltd, China]. The purified DNA was dissolved in 1 × TE buffer and stored at – 20 °C for later use. The PCR amplifications were performed in Bio-Rad S1000 TM Thermal Cycler [Bio-Rad Laboratories, Inc, USA]. The primer sets ITS1/ITS4 (White et al. 1990) were used to amplify the rDNA ITS region, LR5/LR0R (Vilgalys and Hester 1990) were used to amplify the large subunit nuclear ribosomal DNA (nuLSU rDNA) region and EF983F/EF2218R (Örstadius et al. 2015) were used to amplify the translation elongation factor subunit 1 alpha (*Tef1α*) region. The primer sets B36f and B12r (Nagy et al. 2011) were used to amplify the β-tubulin gene (*TUB2*) region. PCRs were performed in a volume of 25 μl consisted of 2 μl of DNA template; 1 μl of (10 μM) per primer; 12.5 μl 2 × Master Mix [Mei5 Biotechnology, Co., Ltd, China]. PCR amplification conditions refer to Bau and Yan (2021). DNA sequences were sequenced by Zhongkexilin Biotechnology, Co., Ltd, Beijing, China.

### ﻿Molecular data analyses

The generated raw reads of the DNA sequences were used to obtain consensus sequences using SeqMan v.7.1.0 in the DNASTAR Lasergene Core Suite software (DNASTAR Inc., Madison, WI, USA). All sequences were aligned using MAFFT v.6 (Katoh and Toh 2010) and trimmed manually using MEGA 6 (Tamura et al. 2013). For phylogenetic analyses, newly-obtained sequences and additional reference sequences of *Candolleomyces* species were included in the dataset of combined ITS-LSU-*Tef1α*-*TUB2* muti-locus DNA (Table 1), with *Psathyrellamultipedata* (Peck) A.H. Sm. (LÖ237-04) used as outgroup. Phylogenetic analyses were performed using PAUP v.4.0b10 for Maximum Parsimony (MP) analysis (Swofford 2003) and MrBayes v.3.1.2 for Bayesian Inference (BI) analysis (Ronquist and Huelsenbeck 2003). ML gene-trees were estimated using the software RAxML 7.4.2 Black Box (Stamatakis 2006; Stamatakis et al. 2008; Zhou and Hou 2019; Zhou et al. 2021).

**Table 1.** Sequences information used in the phylogenetic analysis in this study.

Taxa	Voucher	Locality	ITS	LSU	*β-Tub*	*tef-1α*
*Candolleomycesaberdarensis*	GLM-F116094	Kenya	MH880928	–	–	–
*C.albipes*	DED8340	Sao Tome	KX017209	–	–	–
*C.badhyzensis*	79478 (TAA) Type	Turkmenistan	KC992883	KC992883	–	–
*C.badiophyllus*	SZMC-NL-2347	–	FN430699	FM876268	FN396261	FM897252
*C.cacao*	SFSU DED 8339	Sao Tome	NR148106	–	–	–
*C.cacao*	FP1R4	USA	KU847452	–	–	–
*C.cacao*	MP2R2	USA	KU847436	–	–	–
*C.candolleanus*	LAS73030 Neotype	Sweden	KM030175	KM030175	–	–
*C.cladii-marisci*	CLUF302 Type	Italy	MK080112			
*C.efflorescens*	Pegler2133 (K)	Sri Lanka	KC992941	–	–	–
*C.eurysporus*	GLM-F126263 Type	Germany	MT651560	MT651560	–	–
***C.incanus***	**BJTC Z777 Type**	**China: Beijing**	**ON042759**	**ON042766**	**ON98513**	**ON98508**
***C.incanus***	**BJTC S173**	**China: Beijing**	**ON042760**	**ON042767**	**ON98514**	**ON98509**
*C.leucotephrus*	LÖ138-01 (UPS)	Sweden	KC992885	KC992885	KJ664865	KJ732775
*C.luteopallidus*	Sharp20863 (MICH) Type	USA	KC992884	KC992884	–	–
*C.luteopallidus*	HMJAU5148	China: Jilin	MG734736	MW301084	MW314056	MW314073
*C.secotioides*	UES2918 Type	Mexico	KR003281	KR003282	–	KR003283
*C.singeri*	HMJUA37867	China: Jilin	MG734718	MW301088	MW314059	MW314077
*C.singeri*	HMJAU37877	China: Chongqing	MW301073	MW301091	MW314062	MW314080
*C.subcacao*	HMJAU37807 Type	China: Henan	MW301064	MW301092	MW314063	MW314081
*C.subcacao*	HMJAU37808	China: Henan	MW301065	MW301093	MW314064	MW314082
*C.subcacao*	HFJAU1014	China: Jiangxi	MW559218	–	–	–
*C.subcacao*	HFJAU1274	China: Jiangxi	MW559219	–	–	–
*C.subcacao*	HFJAU1488	China:Anhui	MW559220	–	–	–
***C.subcandolleanus***	**BJTC Z239 Type**	**China: Tianjin**	**ON042755**	**ON042762**	**ON98510**	**ON98505**
***C.subcandolleanus***	**BJTC Z232**	**China: Tianjin**	**ON042756**	**ON042763**	–	–
*C.subminutisporus*	HMJAU37801 Type	China: Hubei	MW301066	MW301094	MW314065	MW314083
*C.subminutisporus*	HMJAU37916	China: Henan	MW301067	MW301095	MW314066	MW314084
*C.subsingeri*	HMJAU37811 Type	China: Jilin	MG734715	MW301097	MW314067	MW314085
*C.subsingeri*	HMJAU37913	China: Jilin	MG734725	MW301098	MW314068	MW314086
*C.sulcatotuberculosus*	GB:LO55-12	–	KJ138422	KJ138422	–	–
*C.sulcatotuberculosus*	HFJAU1515	China: Fujian	MW375696	–	MW382967	MW382965
*C.sulcatotuberculosus*	Chiarello 07-10-2013	–	KJ138423	–	–	–
*C.trinitatensis*	TL9035 (C)	Ecuador	KC992882	KC992882	KJ664863	–
*C.trinitatensis*	ADK4162 (BR)	Togo	KC992886	KC992886	–	–
***C.yanshanensis***	**BJTC Z783**	**China: Beijing**	**ON042757**	**ON042764**	**ON98511**	**ON98506**
***C.yanshanensis***	**BJTC Z110 Type**	**China: Beijing**	**ON042758**	**ON042765**	**ON98512**	**ON98507**
*Candolleomyces* sp.	BAB-4773	India	KP686450	–	–	–
*Candolleomyces* sp.	BAB-5172	India	KR349656	–	–	–
*Candolleomyces* sp.	BAB-4748	India	KR154977	–	–	–
*Candolleomyces* sp.	BAB-4747	India	KR154976	–	–	–
*Candolleomyces* sp.	BAB-5202	India	KT188611	–	–	–
*Psathyrellamultipedata*	LÖ237-04	Sweden	KC992888	KC992888	KJ664867	KJ732777

Notes: The new generated sequences are emphasised in bold.

Maximum Parsimony analysis was performed by a heuristic search option of 1000 random-addition sequences with a tree bisection and reconnection (TBR) algorithm. Maxtrees were set to 1000, branches of zero length were collapsed and all equally parsimonious trees were saved. Other calculated parsimony scores were tree length (TL), consistency index (CI), retention index (RI) and rescaled consistency (RC) (Zhou and Hou 2019).

Maximum Likelihood analysis was performed with a GTR site substitution model (Guindon et al. 2010). Branch support was calculated with a bootstrapping (BS) method of 1000 replicates (Hillis and Bull 1993). Bayesian Inference (BI) analysis, using a Markov Chain Monte Carlo (MCMC) algorithm, was performed (Rannala and Yang 1996). MrModeltest v. 2.3 was used to estimate the best model. Two MCMC chains were run from random trees for 10,000,000 generations and stopped when the average standard deviation of split frequencies fell below 0.01. Trees were saved for each 1000 generations. The first 25% of trees were discarded as the burn-in phase of each analysis. Branches with significant Bayesian posterior probabilities (BPP) were estimated in the remaining trees (Posada and Crandall 1998).

The combined alignment and phylogenetic tree were submitted on TreeBASE (www.treebase.org, study 29579).

## ﻿Result

### ﻿Phylogenetic analyses

**Figure 1.**
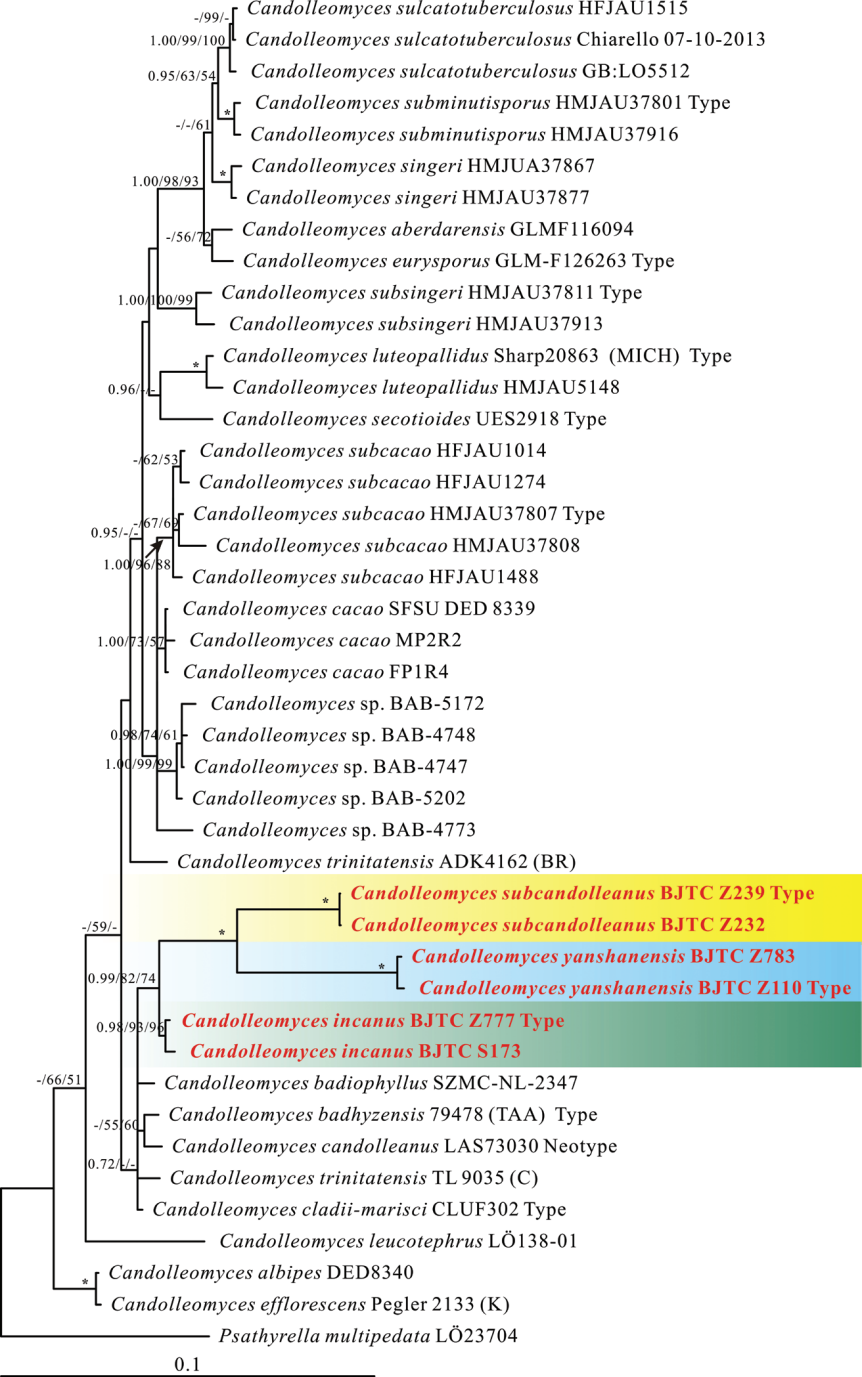
Multi-gene phylogenetic tree obtained from the Bayesian analysis. Numbers above branches are Bayesian posterior probability (pp) values, Maximum Likelihood bootstrap (MLB) and Maximum parsimony bootstrap (MP) values. Asterisks (*) denote branches with pp = 1.00, MLb = 100% and MPb = 100%. Numbers above branches represent strongly and moderately support (pp ≥ 0.95, MLb ≥ 50% and MPb ≥ 50%). The red font indicates the position of the new species.

For the ITS-LSU- *Tef1α*-*TUB2* sequence dataset, a total of 3459 characters including gaps (694 for ITS, 1316 for LSU, 1023 for *Tef1α*, and 426 for *TUB2*) were included in the phylogenetic analysis. Using RAxML, MrBayes and PAUP to construct ML, Bayesian and MP phylogenetic trees, the results show that the topology and branching order were similar and the Bayesian tree is shown in this paper (Fig. 1). The Maximum likelihood analysed was performed with a GTR model. For the Bayesian analyses, the GTR + I + G models were recommended by MrModeltest. The heuristic search using Maximum Parsimony (MP) generated 1000 parsimonious trees (TL = 1168, CI = 0.768, RI = 0.815, RC = 0.232) and branches of zero length were collapsed and all multiple parsimonious trees were saved.

Based on the results, six specimens were assigned to three branches and were described as three new species. The three new species (*Candolleomycesyanshanensis*, *C.subcandolleanus*, *C.incanus*) and a known species (*Candolleomycesbadiophyllus* (Romagn.) D. Wächt. & A. Melzer etc.) clustered together in the phylogenetic tree. The three new species clustered into together (pp = 0.99, MLbs = 82%,MPbs = 74%), but three new species separately formed three subclades with high support value. *Candolleomycesyanshanensis*, *C.subcandolleanus* and *C.incanus* can be distinguished by the phylogenetic tree, sequence base differences and morphological characteristics.

### ﻿Taxonomy

#### ﻿ Candolleomyces yanshanensis

Taxon classificationFungiAgaricalesPsathyrellaceae

C. L. Hou & H. Zhou sp. nov.

1174C24D-B074-5E91-AE43-A6FB466ABD62

 843464

Fig. 2

##### Etymology.

*yanshanensis* referred to the locality where the type specimen was collected.

##### Type.

China, Beijing, Changping District, Beitaizi Village, 40.272906°N, 116.4203°E, alt. 149 m, 14 Aug 2019, coll. X.Y. Shen, H Zhou and R.T. Zhang, BJTC Z110.

**Figure 2.**
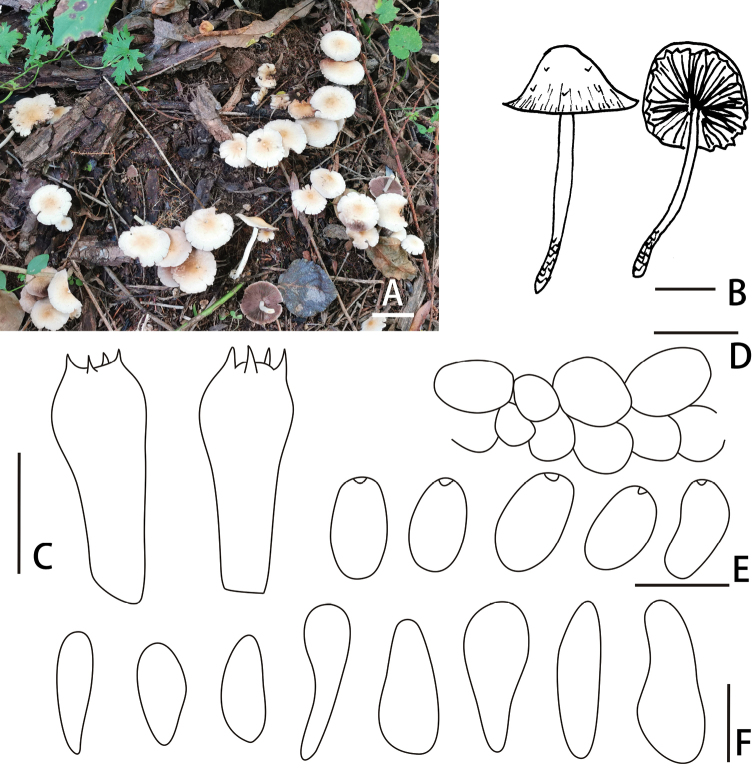
Basidiomata and microscopic features of *Candolleomycesyanshanensis* (BJTC Z110) **A, B** basidiomata **C** basidia **D** pileipellis **E** basidiospores **F** cheilocystidia. Scale bars: 20 mm (**A, B**); 10 μm (**C**); 20 μm (**D**); 5 μm (**E**); 20 μm (**F**).

##### Diagnosis.

*Candolleomycesyanshanensis*, pileus 20–60 mm, flabellate, flattening with age, hygrophanous. Basidiospores 5.8–8.2 × 3.3–5.4 μm, often with germ pore. Subglobose cell, irregular oval, (18) 20–27μm broad.

##### Description.

Pileus 20–60 mm, flabellate, flattening with age, hygrophanous, slightly dirty white (#e3dac9) to pale brown (#deb887). Veil white (#ffffff), fibrils in young, evanescent. Context 1.0–2.0 mm broad at centre, same colour as pileus. Lamellae sparsely to moderately, adnate, slightly dirty white (#e3dac9) to champagne (#fad6a5), edge white (#ffffff) as spores mature. Stipes 50–130 × 3–6 mm, smooth, fibrils on the base, cornsilk (#f0ead6) to white (#ffffff).

Basidiospores 5.8–8.2 × 3.3–5.4 μm, Q = 1.4–2.0, ellipsoid to long ellipsoid, ovoid to ellipsoid, partly triangular at base, dark brown (#b8860b) to brown (#b06500) in water, smooth, abundant, multi-guttules, often with germ pore. Basidia 17–31 × 5.8–7.5 μm, short clavate, hyaline, 4-spored. Cheilocystidia 22–35 (40) × 8–11 (15) μm, irregular utriform or claviform, apex obtuse or broadly obtuse or often subcapitate, rarely with deposits. Pileipellis consists of 2–3 cells deep layer of irregular subglobose cell, irregular oval, (18) 20–27μm broad.

##### Habit and habitat

. Clumped on the ground with rich humus in broad-leaved forests or broad-leaved shrubs.

##### Additional specimen examined.

China, Beijing, Changping District, Tailing, 40.327397°N, 116.21916°E, alt. 172 m, 17 Aug 2020, coll. X.Y. Shen, H Zhou and X.B. Huang, BJTC Z783.

#### ﻿ Candolleomyces subcandolleanus

Taxon classificationFungiAgaricalesPsathyrellaceae

C. L. Hou & H. Zhou sp. nov.

DEB32C82-897C-5985-96E9-A8C8960566D7

 843466

Fig. 3

##### Etymology.

*subcandolleanus* referred to its morphological similarity to *Candolleomycescandolleanus* (Fr.) D. Wächt. & A. Melzer.

**Figure 3.**
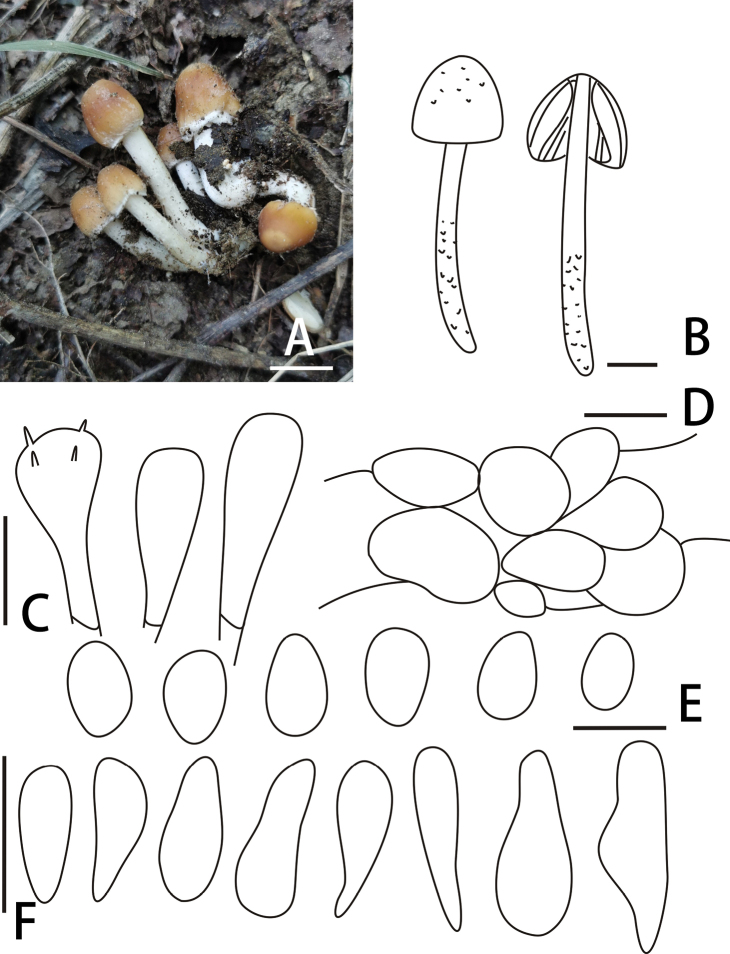
Basidiomata and microscopic features of *Candolleomycessubcandolleanus*. (BJTC Z239) **A, B** basidiomata **C** basidia **D** pileipellis **E** basidiospores **F** cheilocystidia. Scale bars: 10 mm (**A, B**); 10 μm (**C**); 20 μm (**D**); 5 μm (**E**); 20 μm (**F**).

##### Type.

China, Tianjin, Jizhou District, Sanjiebei, 40.227984°N, 117.43354°E, alt. 235 m, 17 Aug 2019, coll. X.Y. Shen, H. Zhou and R.T. Zhang, BJTC Z239.

##### Diagnosis.

*Candolleomycessubcandolleanus*, pileus 5–20 mm. Basidiospores 5.5–6.7 × 3.2–4.5 μm, germ pore absent. Cheilocystidia 21–28 (30) × 8–12 (15) μm. Subglobose cell, irregular oval or long oval, (13) 16–25 μm broad.

##### Description.

Pileus 5–20 mm, campanulate to conical, smooth, fibrils in young, evanescent, brown (#b06500) to golden brown (#996515). Veil white (#ffffff), fibrils in young, evanescent. Context 0.2–0.5 mm broad at centre, same colour as pileus. Lamellae moderately to normally, adnate, slightly dirty white (#e3dac9) to white (#ffffff), edge white (#ffffff) as spores mature. Stipes 20–60 × 1–3 mm, smooth, fibrils on the base, cornsilk (#f0ead6) to white (#ffffff).

Basidiospores 5.5–6.7 × 3.2–4.5 μm, Q = 1.4–2.0, ellipsoid to ovoid, pale cream (#fffff0) to pale lemon (#fffacd) in water, smooth, multi-guttules, germ pore absent. Basidia 18–27 × 5–10 μm, short clavate, hyaline, 4-spored. Cheilocystidia 21–28 (30) × 8–12 (15) μm, utriform or claviform, apex obtuse or broadly obtuse or often subcapitate, rarely with deposits. Trama of gills irregular. Pileipellis consists of irregular subglobose cell, irregular oval or long oval, (13) 16–25 μm broad.

##### Habit and habitat.

Clumped on the ground with rich humus in broad-leaved forests or broad-leaved shrubs.

##### Additional specimen examined.

China, Tianjin, Jizhou District, Huangyaguan Great Wall, 40.245615°N, 117.44047°E, alt. 235 m, 17 Aug 2019, coll. X.Y. Shen, H. Zhou and R.T. Zhang, BJTC Z232.

#### ﻿ Candolleomyces incanus

Taxon classificationFungiAgaricalesPsathyrellaceae

C. L. Hou & H. Zhou sp. nov.

17C7B0AF-0403-5D3A-B527-B7EC229C51B5

 843465

Fig. 4

##### Etymology.

*incanus* referred to the basidiomata appears incanus.

##### Type.

China, Beijing, Changping District, Sidaohe Village, 40.246374°N, 116.4406°E, alt. 114 m, 16 Aug 2020, coll. X.Y. Shen, H Zhou and X.B. Huang, BJTC Z777.

**Figure 4.**
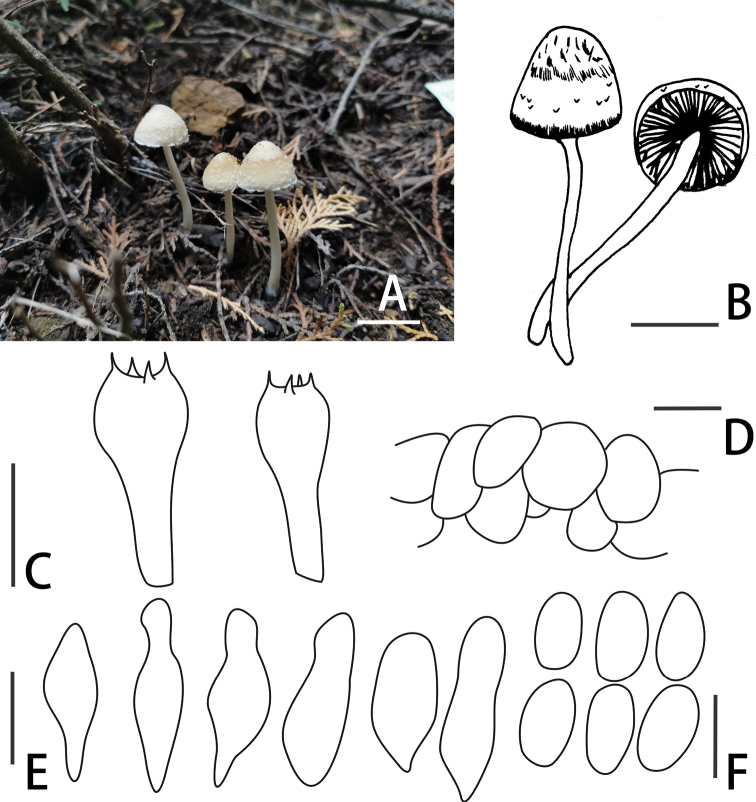
Basidiomata and microscopic features of *Candolleomycesincanus* (BJTC Z777) **A, B** basidiomata **C** basidia **D** pileipellis **E** cheilocystidia **F** basidiospores. Scale bars: 20 mm (**A, B**); 10 μm (**C**); 20 μm (**D, E, F**).

##### Diagnosis.

*Candolleomycesincanus*, pileus 5–25 mm, hemispherical to conical. Basidiospores 6.0–7.0 × 3.2–4.5 μm. Stipe 40–70×4–6 mm, smooth, germ pore absent. Subglobose cell, irregular oval or long oval, (22) 25–32 μm broad.

##### Description.

Pileus 5–25 mm, hemispherical to conical, hygrophanous, incanus (#f2f3f4) to nude (#fdf5e6). Veil white (#ffffff), fibrils in young, evanescent. Context 0.5–1.0 mm broad at centre, same colour as pileus. Lamellae moderately to normally, adnate, off-white (#f2f3f4) to white (#ffffff), edge white (#ffffff) as spores mature. Stipes 40–70 × 4–6 mm, smooth, hygrophanous, cornsilk (#f0ead6) to white (#ffffff).

Basidiospores 6.0–7.0 × 3.2–4.5 μm, Q = 1.4–1.9, ellipsoid, floral white (#fffaf0) to dark yellow (#eedc82) in water, smooth, abundant, multi-guttules, germ pore absent. Basidia 15–20 × 5–8 μm, short clavate, hyaline, 4-spored. Cheilocystidia 17–27 (31) × 7–11 (13) μm, utriform, apex obtuse or broadly obtuse or often subcapitate, rarely with deposits. Trama of gills irregular. Pileipellis consisted of irregular subglobose cell, irregular oval or long oval, (22) 25–32 μm broad.

##### Habit and habitat.

Clumped on the ground with rich humus in deciduous broad-leaved or deciduous coniferous forests.

##### Additional specimen examined.

China, Beijing, Yanqing District, Yudu Mountain, 40.54399°N, 115.893984°E, alt. 860 m, 12 Sep 2018, coll. C.L. Hou, H Zhou and J.Q. Li, BJTC 646.

## ﻿Discussion

In this study, three new species were identified by morphology and phylogeny. It is very interesting that the three new species *C.yanshanensis*, *C.subcandolleanus* and *C.incanus* formed a stronger supported clade and they clustered with *Candolleomycesbadiophyllus* (Romagn.) D. Wächt. & A. Melze, *Candolleomycescandolleanus*, *Candolleomycesbadhyzensis* (Kalamees) D. Wächt. & A. Melzer, *Candolleomycestrinitatensis* (R.E.D. Baker & W.T. Dale) D. Wächt. & A. Melzer and *Candolleomycescladii-marisci* (Sicoli, N.G. Passal., De Giuseppe, Palermo & Pellegrino) J.Q. Yan together in the phylogenetic tree. In addition, three new species were weakly sister to the known species *C.badiophyllus* in the phylogenetic tree.

*Candolleomycesyanshanensis* and *C.subcandolleanus* are different in macroscopic morphology of basidiomata. *Candolleomycesyanshanensis* is lighter in pileus colour and *C.yanshanensis* has larger spores (5.8–8.2 × 3.3–5.4 vs. 5.5–6.7×3.2–4.5 μm) and longer cheilocystidia (22–35 × 8–11 vs. 21–28 × 8–12 μm) than those of *C.subcandolleanus*. Moreover, *C.yanshanensis* spores often have a germ pore. *Candolleomycessubcandolleanus* is very easily confused with *C.candolleanus* in the field because of their similar macroscopic characteristics. In particular, two species in these sections possess the combined characteristics of small basidiomata. *C.candolleanus* is the type species of *Candolleomyces*, with early studies on this species being based on the number of pleats and other characteristics, but this also led to confusion in the identification of this species. *Candolleomycessubcandolleanus* can be distinguished from *C.candolleanus* by the smaller spores (5.5–6.7 × 3.2–4.5 vs. 7–9 × 4–5 μm) (Kits van Waveren 1980; Breitenbach and Kränzlin 1995; Mifsud 2017).

*Candolleomycesincanum*, *C.badiophyllus*, *C.candolleanus* and *C.badhyzensis* are close to each other in the phylogenetic tree. However, the four species show significant differences in morphology. These species can be distinguished as follows: *C.incanus* has smaller and narrower spores (6.0–7.0 × 3.2–4.5 μm), whereas *C.candolleanus*, *C.badhyzensis* and *C.badiophyllus* have larger spores (Spores of *C.candolleanus* were 7.0–9.0 × 4.0–5.0 μm, spores of *C.badhyzensis* were 10.2–11.5 × 5.5–6.5 μm, spores of *C.badiophyllus* were 10–14 × 5–6 μm). In addition, *C.incanus* has smaller cheilocystidia (17–27 × 7–11 vs. 34–51 × 10–15 μm) than those of *C.badhyzensis* (Kalamees 1981; Kasik et al. 2004; Wächter and Melzer 2020).

Except for morphological differences, the three new species in this study can also be distinguished by sequence similarity. *Candolleomycesyanshanensis* (BJTC Z110) can be distinguished, based on nucleotide differences in ITS, LSU, *Tef1α* and *TUB2* loci from *C.subcandolleanus* (BJTC Z239) (sequence base similarity 93% in ITS, 100% in LSU, 99% in *Tef1α* and 98% in *TUB2*); *C.yanshanensis* (BJTC Z110) can be distinguished, based on nucleotide differences from *C.incanus* (BJTC Z777) (sequence base similarity 80% in ITS, 99% in LSU, 99% in *Tef1α* and 96% in *TUB2*); *C.subcandolleanus* (BJTC Z239) can be distinguished, based on nucleotide differences from *C.incanus* (BJTC Z777) (sequence base similarity 81% in ITS, 99% in LSU, 99% in *Tef1α* and 98% in *TUB2*). It can also be found that the ITS loci have a greater degree of differentiation for the species in *Candolleomyces*, Nevertheless, LSU and *Tef1α* were more conservative for the genus.

According to the research of Wächter and Melzer (2020), the species of *Candolleomyces* may be more abundant than previously thought and better delimitation of species boundaries is required. While the boundaries of some species are disputed, the number of new taxa is steadily increasing (Sicoli et al. 2019; Büttner et al. 2020; Bau and Yan 2021). However, the continued discovery of clear boundaries in new taxa like this study enhances our comprehension of species in this genus.

It is considered that the natural growth of *Candolleomyces* may be related to precipitation. However, the investigation and specimen collection in this study were carried out in the rainy season in July to August, with no collection in other periods. Therefore, more species of *Candolleomyces* might be expected in Yanshan Mountains.

## Supplementary Material

XML Treatment for Candolleomyces yanshanensis

XML Treatment for Candolleomyces subcandolleanus

XML Treatment for Candolleomyces incanus
